# Bioprospecting Honey-Derived Microorganisms for the Biological Control of Phytopathogens

**DOI:** 10.3390/microorganisms14010224

**Published:** 2026-01-18

**Authors:** Patrícia Perina de Oliveira, Giovanna Felette de Paula, Katherine Bilsland Marchesan, Luiza Rodrigues de Souza, José Fhilipe de Miranda da Silva, João Gabriel Elston, Henrique Marques de Souza, Elizabeth Bilsland

**Affiliations:** 1 Synthetic Biology Laboratory, Department of Structural and Functional Biology, Institute of Biology, Universidade Estadual de Campinas, Campinas 13084-864, SP, Brazil; p156922@dac.unicamp.br (P.P.d.O.); g204730@dac.unicamp.br (G.F.d.P.); j272430@dac.unicamp.br (J.F.d.M.d.S.); 2 Instituto Federal de Educação, Ciência e Tecnologia de São Paulo, Câmpus Campinas, Campinas 13059-581, SP, Brazil; katherine.b@aluno.ifsp.edu.br (K.B.M.); luiza.souza@aluno.ifsp.edu.br (L.R.d.S.); 3 Brazilian Laboratory on Silencing Technologies, Department of Biochemistry and Histology, Institute of Biology, Universidade Estadual de Campinas, Campinas 13083-864, SP, Brazil; j.gabriel_elston@hotmail.com (J.G.E.); hmsouza@unicamp.br (H.M.d.S.)

**Keywords:** biological control, phytopathogens, sustainable agriculture, honey, *Bacillus*

## Abstract

Microbial biological control agents are a sustainable alternative to synthetic pesticides, yet their widespread application is limited by a lack of environmental resilience of commercial products. To address this, we exploited honey—a stringent ecological niche—as a reservoir for stress-tolerant bacteria. In this study, the bioprospection utilizing five types of commercially available honeys yielded a collection of 53 bacteria and 10 fungi. All bacterial isolates were evaluated for antimicrobial activity against a laboratory-standard bacterium and yeast, and six economically relevant phytopathogenic microorganisms. Initial screening with standard laboratory organisms proved to be an efficient method to detect strains with antimicrobial potential, correlating significantly with further phytopathogen inhibition (Spearman’s r = 0.4512, *p* = 0.0005). Two promising strains, M2.7 and M3.18, were selected for quantitative dual-culture assays along with molecular identification using 16S rDNA and *gyrA* gene sequencing, classifying them as *Bacillus velezensis*. These strains exhibited high inhibitory effects against the pathogens (*p* > 0.001), often with equivalent efficacy to the commercial biocontrol strain, and also induced significant phytopathogen hyphal deformities, such as increased septation and swelling. These findings support honey as a viable source of robust biocontrol agents, offering a sustainable strategy to substitute or complement current agrochemicals.

## 1. Introduction

Agriculture is a cornerstone of human civilization, yet its capacity to ensure global food security is under unprecedented strain from bacterial and fungal infections exacerbated by climate change and antimicrobial resistance (AMR) [1,2,3,4]. The Food and Agriculture Organization (FAO) estimates that plant diseases cost the global economy around $220 billion annually, roughly 10% to 16% of worldwide agricultural output [2,5]. While agrochemicals and biotechnological advances such as the development of genetically modified organisms (GMOs) have been a key pillar in amplifying agricultural production and increasing the global food supply [1,6], the accelerated growth of the world population, which is expected to reach 9.7 billion people by 2050, requires even greater efficiency [1].

However, conventional systems relying on heavy chemical inputs face diminishing returns and rising regulatory pressure due to socio-environmental impacts [7,8]. In Brazil, a top global consumer of chemical pesticides in agriculture (337 kilotons in 2020), the excessive use of these products has been linked to ecosystem damage, soil and water pollution, and adverse health effects for rural populations [5,6,9]. This reliance on antibiotics and antifungals has greatly contributed to the rise of AMR, compromising treatments in both agriculture and medicine [8,10,11,12]. Evidence suggests a correlation between agricultural antibiotic use and the emergence of resistant human pathogens via horizontal gene transfer [3,4,11]. Although the volume of antibiotics used in plant protection is lower than in clinical settings, their impact is significant as they are frequently grouped with fungicides due to their broad-spectrum activity [3,11]. These resistance patterns complicate the management of key phytopathogens. Bacterial genera such as *Xanthomonas, Erwinia*, and *Xylella* contribute to annual crop losses of up to 5 billion euros [11,13,14,15]. A notable example is *X. citrus,* the agent of citrus canker, which drastically reduces the market value of citrus crops [15,16,17].

Similarly, invasive fungal infections are a major component of the AMR crisis, with resistance to systemic drugs often being traced to environmental exposure to agricultural fungicides [12,18,19]. Key fungal threats include *Sclerotinia sclerotiorum* (Lib.) de Bary, the agent of “white mold,” which can cause complete crop loss in economically important plants like soybeans and beans [20], and the ubiquitous soil pathogen *Rhizoctonia solani* JG Kühn [Teleomorph: *Thanatephorus cucumeris* (AB Frank) Donk], which causes “damping off” in crops like rice and potato [21,22]. The genus *Fusarium* is equally devastating, with species like *F. verticillioides* (Sacc.) and Nirenberg (*syn*. *F. moniliforme*, Sheldon) responsible for maize rot and the production of mycotoxins [23,24,25], and *F. oxysporum*, known for causing devastating “Fusarium wilt” in banana and tomato, capable of annihilating entire plantations [26,27,28]. Furthermore, in post-harvest storage, “gray mold,” caused by *Botrytis cinerea* Pers., leads to significant commercial losses in fruits like strawberries and grapes [29,30,31]. Together, these pathogens, coupled with climate change and growing AMR, create an escalating risk to global food security [4,32,33,34].

Given these concerns, current agricultural methods must be complemented by sustainable approaches [4,6,9,32]. An alternative to chemical pesticides that is gaining ground worldwide is biological control strategies, which are compatible with Integrated Pest Management (IPM) and organic farming. In this scenario, bioprospecting microorganisms is a pivotal strategy to address this global demand for sustainability [35,36]. Historically, soil-based bioprospecting has led to the identification of most known antibiotics, but the high rediscovery rate has shifted focus towards less conventional sources of microorganisms [33,35,36,37]. One promising avenue involves investigating microorganisms that live in symbiosis with insects, as these unique environments may harbor previously unexamined biosynthetic pathways capable of producing novel bioactive compounds [38,39,40].

Beyond agricultural productivity, the search for resilient biocontrol agents aligns with the One Health framework, which recognizes the fundamental link between environmental, animal, and human health. As highlighted in recent studies, adopting biopesticides is a vital strategy for promoting planetary health, as it reduces the chemical footprint in ecosystems and mitigates the risks associated with AMR [41,42]. *Bacillus* species are notable for their resilience, ability to enhance plant growth, and their suppression of phytopathogens through various mechanisms, including the production of specialized metabolites [35,36,43,44]. Identifying new strains with unique biocontrol properties is critical for addressing the intensification of agricultural practices [4,42,45]. Our study investigates honey as an unconventional, high-osmolarity source of microbial diversity, potentially harboring resilient biocontrol agents better suited for field stress than traditional soil isolates [46]. This work contributes to the growing body of research aimed at developing sustainable agricultural practices aligned with global food security goals.

## 2. Materials and Methods

### 2.1. Biological Materials

The biological material used was isolated from five types of commercial honey (Table 1). Each honey was diluted (1:3, *v*/*v*) in sterile water, followed by a 1:1000 (*v*/*v*) dilution. Isolation was performed by plating 100 µL of these dilutions on solid LB medium (tryptone 10 g·L^−1^, yeast extract 5 g·L^−1^, NaCl 10 g·L^−1^, agar 10 g·L^−1^), TMA (casein peptone 7.5 g·L^−1^, meat peptone 7.5 g·L^−1^, corn starch 1 g·L^−1^, K_2_HPO_4_ 4 g·L^−1^, KH_2_PO_4_ 1 g·L^−1^, NaCl 5 g·L^−1^, agar 12 g·L^−1^), TSA (soybean meal peptone 5 g·L^−1^, NaCl 5 g·L^−1^, agar 15 g·L^−1^), and HS medium (peptone 5 g·L^−1^, yeast extract 5 g·L^−1^, disodium phosphate 2.7 g·L^−1^, citric acid 1.5 g·L^−1^, glucose 20 g·L^−1^, agar 15 g·L^−1^), incubating at 30 °C for 2 to 5 days. The isolated colonies were subsequently transferred to fresh media for stock preparation and further testing. The 1:1000 dilution was used as a low-load plating control, with no colonies being detected at this dilution.

### 2.2. Disk Diffusion Assay

To assess whether bacteria isolated from commercial honey samples showed antimicrobial activity, inhibition tests were conducted with these microorganisms against *Escherichia coli* DH5α and *Saccharomyces cerevisiae* BY4741/yEP-GAP-mCherry [47] separately. Furthermore, six phytopathogens, namely *Xanthomonas citri*, *Fusarium verticillioides*, *F. oxysporum*, *Sclerotinia sclerotiorum*, *Rhizoctonia solani*, and *Botrytis cinerea*, were also used in disk diffusion assays to assess the inhibition capacity of the bacterial isolates. In these experiments, as the objective was to pre-select the bacterial isolates with antimicrobial production potential, standardized inoculum densities were not employed at this stage. Therefore, the purpose of this assay was to evaluate the inhibition potential of the isolates qualitatively.

**Figure 1:**
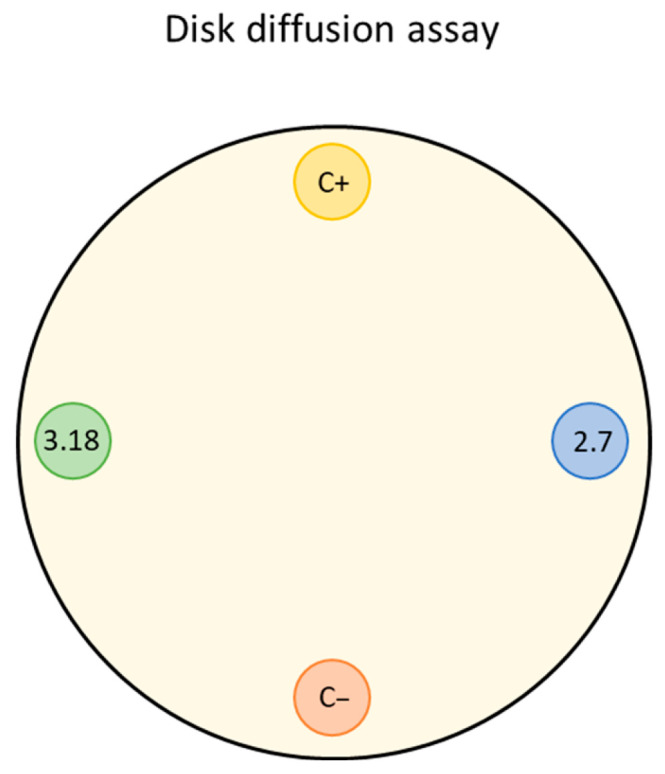
Schematic representation of the disk diffusion assay used to assess qualitative antimicrobial activity of bacterial isolates.

The isolated microorganisms, the phytopathogenic bacteria *Xanthomonas citri* and the strain *Escherichia coli* DH5α, were inoculated individually into liquid LB medium; the yeast *Saccharomyces cerevisiae BY4741/yEP-GAP-mCherry* was inoculated into YNB-URA (Yeast Nitrogen Base without amino acids 6.7 g·L^−1^, glucose 20 g·L^−1^, and dropout supplement without uracil) liquid medium. The samples were incubated at 30 °C for 24 h under constant agitation. A disk diffusion assay (Figure 1) was then performed on solid LB and YNB-URA media, and these plates were incubated for 2 days at 30 °C. After this period, the inhibition halos of bacterial and yeast growth were evaluated.

For the fungal inhibition assays, the phytopathogens *Fusarium verticillioides*, *F. oxysporum*, *Sclerotinia sclerotiorum*, *Rhizoctonia solani*, and *Botrytis cinerea* were tested using a method similar to an antibiogram. The phytopathogen was placed on the plate, with eight equidistant bacterial strains arranged around it, allowing for the observation of inhibition zones (halos) to assess qualitative inhibition. The phytopathogens were cultured on PDA medium (20 g·L^−1^ dextrose, 4 g·L^−1^ potato infusion, and 15 g·L^−1^ agar) for 7 days at their optimal growth temperature. Disks with a 10 mm diameter, or a sample taken with a loop, were collected from the outermost region of the fungal colonies and either placed in the center of a fresh PDA plate or spread across the entire surface. Then 1 µL of each bacterial overnight suspension was transferred to autoclaved filter paper discs surrounding the phytopathogen; these plates were incubated for 7 days at 30 °C. The evaluation was based on the presence of inhibition zones (halos) around the bacterial colonies.

### 2.3. Fungal Inhibition and Morphological Analysis

The two strains that showed the best results in the initial halo assays (M2.7 and M3.18) were selected for a quantitative disk diffusion test. The evaluation was based on comparing the radius of the fungal colonies on control plates (Dc), without the bacterial isolates, and on test plates (Dt), with the isolates, using the formula described by Edgington et al. [48]: I% = [(Dc − Dt)/Dc] × 100. The growth inhibition radius of the phytopathogenic fungi was measured using the program ImageJ version 1.54p.

**Figure 2:**
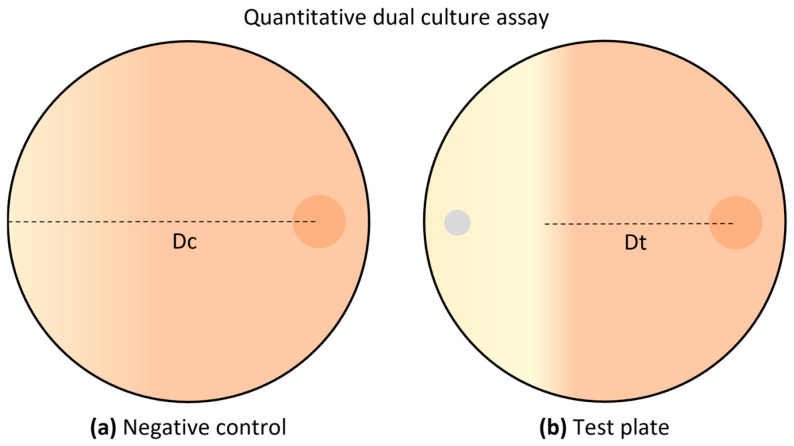
Schematic representation of the dual culture assay used to assess fungal growth inhibition: (**a**) negative control plates involved plating the phytopathogenic fungi alone; (**b**) dual culture of the phytopathogenic fungi against bacterial strains. For the dual culture, the bacterial isolate (represented by a gray disk) was inoculated on one side of the plate, while the phytopathogenic fungi (represented by an orange disk) was added to the opposite side. Dc—distance of fungal growth in the control plate, which is considered as 100% growth; Dt—distance of fungal growth in the test plate, used to calculate the percentage of growth inhibition.

For the test plates (Figure 2b), a 10 mm diameter disk containing the phytopathogenic fungi was positioned 1 cm from the edge of each plate, which had fresh PDA medium. On the same plate, 1 µL of each bacterial isolate at an optical density of 0.01 was inoculated on the opposite side, also 1 cm away from the edge of the Petri dish. Regarding the control plates (Figure 2a), no bacterial isolates were applied, and only the disk with the phytopathogenic fungi was positioned. All quantitative disk diffusion tests were performed in triplicate. These plates were incubated for 7 days at 25 °C, and photographs were taken periodically every 24 h.

The morphological analysis assay was performed using a dissection microscope (Singer Instruments, Watchet, UK) to visualize the morphology of the phytopathogenic fungi’s hyphal structures, indicating once again that the bacterial isolates had antibiosis activity against those pathogens. The plates utilized in this experiment were the same as the ones made to quantify the growth inhibition of the fungi, after 7 days of incubation for all fungi, except *R. solani* and *S. sclerotiorum*, which were analyzed after 2 and 3 days of incubation, respectively. The hyphal morphology on control plates was considered expected and naturally occurring, while the different hyphal morphology occurring on test plates was an indicator of the bacterial isolate’s antimicrobial action.

### 2.4. Statistical Analysis

All data processing and statistical analyses were performed using GraphPad Prism software (Version 10.1.2, GraphPad Software, San Diego, CA, USA). The significance level was set at *p* < 0.05. Statistical significance in all presented figures is denoted by asterisks (*). Differences were considered statistically significant when the *p*-value resulting from the respective tests fell below 0.05. The convention employed is as follows: a single asterisk (*) indicates significance (0.01 ≤ *p* < 0.05); two asterisks (**) denote high significance (0.001 ≤ *p* < 0.01); three asterisks (***) represent extreme significance (*p* < 0.001); and four asterisks (****) represent maximum statistical significance (*p* < 0.0001).

#### 2.4.1. Proxy System Validation and Correlation Analysis

The predictive capacity of the proxy organisms (*E. coli* and *S. cerevisiae*) was validated using a combined statistical approach. The correlation between the proxy inhibition score (ranging from 0 to 2) and the total number of inhibited phytopathogens was assessed using Spearman’s Rank Correlation test, reporting the coefficient (r_s_) and the two-tailed *p*-value. To assess the median trend, the Kruskal–Wallis test, followed by a test for linear trend, was applied to confirm a monotonic increase in the median number of inhibited phytopathogens across the proxy score groups. Furthermore, the ability of the proxy score to discriminate between truly active and non-active isolates was evaluated by constructing a receiver operating characteristic (ROC) curve. Predictive power was quantified by the area under the curve (AUC).

#### 2.4.2. Assessment of Antagonistic Activity

Raw measurements of hyphal length and percentage inhibition data were first assessed for normality. Given the nature of the data and non-normal distribution, the Kruskal–Wallis test was employed to compare the median antagonistic activities of the test isolates and the positive control (*B. subtilis* QST713) against the untreated control group, following a significant global result, the Mann–Whitney U test or Kruskal–Wallis followed by Dunn’s multiple-comparison test was utilized post hoc to determine the specific pairwise differences.

### 2.5. Sequencing and Molecular Identification

The selected strains from the halo inhibition assays were cultivated in liquid medium LB under constant agitation for 48 h at 30 °C. For molecular identification, genomic DNA (gDNA) was extracted using 750 µL of each culture, transferred to 1.5 mL tubes, and centrifuged at 15,000 rpm for 2 min. The resulting pellet was separated, and the supernatant was discarded. The pellet was then resuspended in 100 µL of a solution containing 200 mM lithium acetate (LiAc) and 1% sodium dodecyl sulfate (SDS), followed by incubation at 70 °C for 15 min. Afterward, 350 µL of 100% ethanol was added to precipitate the DNA, and the mixture was centrifuged at 15,000 rpm for 3 min. The supernatant was discarded, and the DNA pellet was washed with 200 µL of 70% ethanol to remove impurities. The tube was centrifuged at 15,000 rpm for 3 min. The supernatant was discarded, and the DNA pellets were allowed to air-dry. Once dried, the pellets were resuspended in 150 µL of ultrapure water. This extracted genomic DNA was used for the amplification and isolation of the 16S ribosomal RNA gene region and the *gyrA* gene region, which encodes the A subunit of DNA gyrase.

The amplification of the 16S rRNA and *gyrA* genes was carried out using the primers listed in Table 2. The amplification protocol used 0.5 µL of 10 µM primer solutions, 1.0 µL of gDNA from each strain, 10 µL of Taq polymerase buffer (PCRBiosystems, London, UK), and 0.25 µL of Taq polymerase (PCRBiosystems), and the final volume was adjusted to 50 µL with ultrapure water. The PCR reaction was performed using an thermocycler (Eppendorf, Hamburg, Germany) .

The amplification of the 16S rRNA was performed using the following cycles: 95 °C for 1 min, 95 °C for 15 s, 60 °C for 15 s, 72 °C for 30 s, repeated for 40 cycles, and a final hold at 4 °C. The amplification of the *gyrA* gene region with the primer gyrA1 was done at 95 °C for 2 min, 30 cycles of 95 °C for 15 s, 52 °C for 15 s, and 72 °C for 30 s, followed by a final extension of 72 °C for 1 min and a 4 °C final hold. The PCR reaction with the primer pair gyrA2 followed the protocol: 95 °C for 2 min; 35 cycles at 95 °C for 15 s, 50 °C for 30 s, and 72 °C for 30 s, and the final extension at 72 °C for 1 min.

After the reaction, a 1% agarose gel in TAE buffer (4.84 g·L^−1^ tris base, 1.14 mL·L^−1^ glacial acetic acid, 2 mL·L^−1^ 0.5 M EDTA) was prepared and transferred to an electrophoresis chamber with TAE buffer. The amplified DNA was prepared with a loading buffer to increase density and blue dye, and this solution was loaded into the wells. The gel was run at 100 V for 30 min, and the results were photo-documented using a ultraviolet transilluminator (Bio-Rad Laboratories, Hercules, CA, USA).

The PCR products were purified using the GENECLEAN Turbo for PCR kit (MP Biomedicals, Irvine, CA, USA) and sent for Sanger sequencing at the Human Genome and Stem Cell Research Center (Institute of Biosciences—USP). The Sanger sequencing results were analyzed using the NCBI BLAST database using the BLASTn algorithm (https://blast.ncbi.nlm.nih.gov/Blast.cgi; accessed on 14 November 2024) [49] and the EZBioCloud 16S database (https://www.ezbiocloud.net/; accessed on 14 November 2024), generating a closeness percentage between the honey isolates and other strains deposited in the database.

## 3. Results

### 3.1. Bioprospecting

Honey is generally considered an inhospitable environment for microbial growth due to its high osmolarity; therefore, we plated honey samples onto different culture media with the expectation of encountering none or very few colony-forming units. Surprisingly, our experiments yielded a large variety of microorganisms.

**Figure 3:**
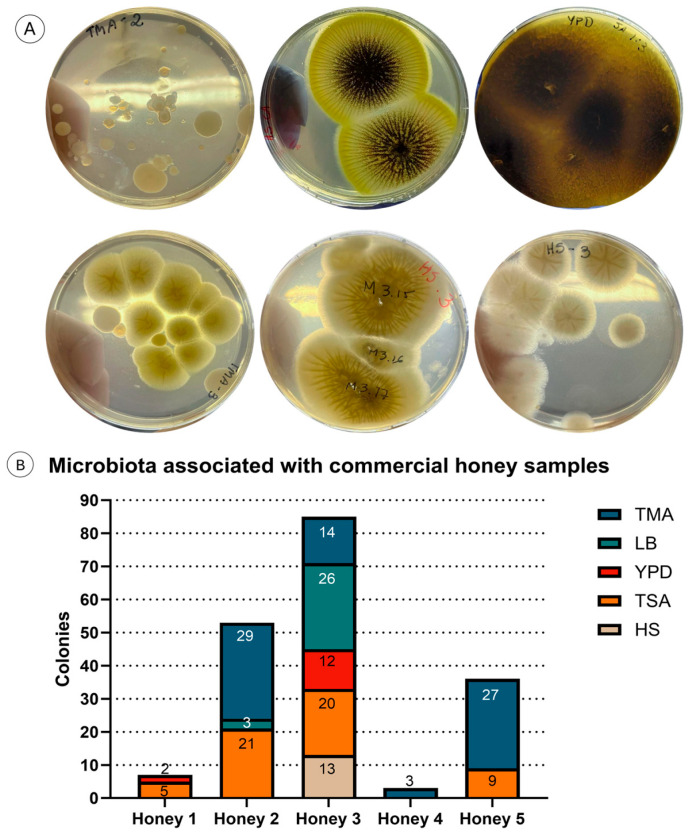
Results of bioprospecting: (**A**) plates illustrating the diversity of microorganisms present in honey samples which were diluted, plated onto different media and incubated at 30 °C for 2–5 days; (**B**) quantification of fungal and bacterial colonies derived from 100 μL of 5 different honey samples (Honey 1–5) plated onto TMA, LB, YPD, TSA or HS media. Honey 1—*Apis mellifera* honey sachet; Honey 2—*Tetragonisca* sp.; Honey 3—*Apis mellifera* with comb; Honey 4—*Apis mellifera* honeydew; Honey 5—*Apis mellifera*.

Different commercial honey samples were used, with 100 μL of honey plated onto each media, resulting in growth of a variable number of microbial colonies, where samples derived from honey produced by the native Brazilian stingless bee *Tetragonisca* sp. (honey 2) and honey from *Apis mellifera* with comb fragments (honey 3) yielded a higher number of associated microorganisms (Figure 3). It is important to highlight the fact that the fungi or bacteria could have been present in the honey whilst in the hive, or they could have been acquired during harvesting or processing, with changes in the population during storage [50,51]; hence, we cannot draw conclusions on the bee-associated microbiota based on our results.

A total of 7 colonies were formed on plates inoculated with Honey 1, with 2 from YPD and 5 from TSA medium. Honey 2 yielded 53 colonies, including 29 from TMA, 3 from LB, and 21 from TSA. Honey 3 had the highest count, with 85 microorganisms forming colonies: 14 from TMA, 26 from LB, 12 from YPD, 20 from TSA, and 13 from HS medium. Honey 4 yielded only 3 colonies, all from TMA medium. Honey 5 yielded 36 colonies, with 27 from TMA and 9 from TSA (Figure 3B). Several colonies formed were phenotypically indistinguishable; therefore, we isolated all unique and up to 2 similar colonies from each honey–media combination, amounting to 53 bacterial and 10 fungal strains. At that moment, we focused our efforts on the potential of the bacterial strains.

### 3.2. Results of Disk Diffusion Assay

**Figure 4:**
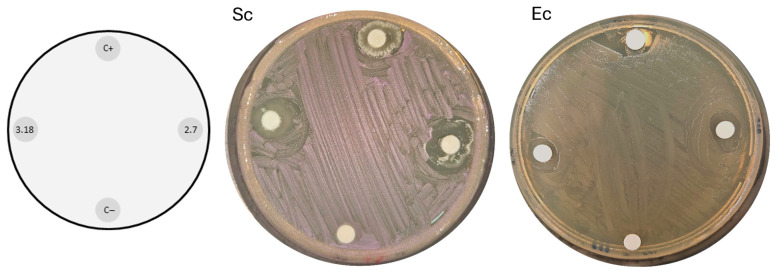
Disk diffusion assays with bacterial isolates against *Escherichia coli* DH5α (Ec) and *Saccharomyces cerevisiae* BY4741 (Sc) showing inhibition halos, indicating their antimicrobial activity. At the bottom of each plate, the negative control *Bacillus subtilis* 168 was inoculated; to the right, isolate M2.7; at the top, the positive control *Bacillus velezensis* QST713; and to the left, isolate M3.18. This experimental setup was reproduced for all 53 bacterial isolates.

Plates derived from our bioprospecting showed that several organisms inhibited the growth of neighboring colonies; therefore, we performed disk diffusion assays of all of our isolates against the standard laboratory strains *Escherichia coli* DH5α (Gram-negative bacterium) and *Saccharomyces cerevisiae* BY4741/yEP-GAP-mCherry (Figure 4).

**Figure 5:**
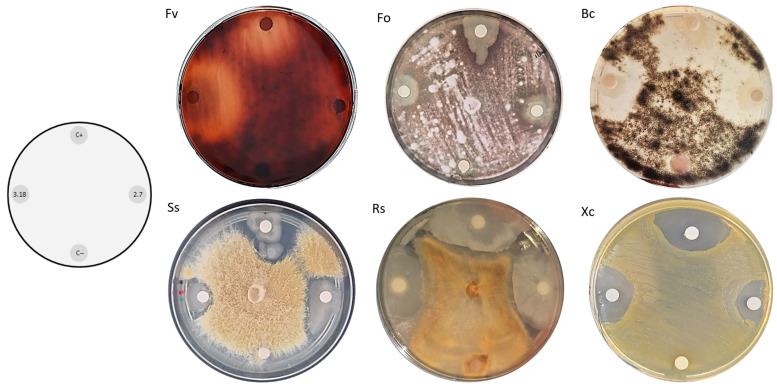
Disk diffusion assays with 2 bacterial isolates (M2.7 and M3.18) against different phytopathogens, showing clear inhibition halos, which indicates their antimicrobial activity. At the top of each plate, the positive control *Bacillus velezensis* QST713 was inoculated; to the right, isolate M2.7; at the bottom, the negative control *Bacillus subtilis* 168; and to the left, isolate M3.18. The tested phytopathogens include: Fv—*Fusarium verticillioides*; Fo—*F. oxysporum*; Bc—*Botrytis cinerea*; Ss—*Sclerotinia sclerotiorum*; Rs—*Rhizoctonia solani*; Xc—*Xanthomonas citri*.

Encouraged by our preliminary results, we acquired 6 phytopathogens, namely the fungi *Rhizoctonia solani*, *Sclerotinia sclerotiorum*, *Fusarium verticillioides*, *Fusarium oxysporum*, and *Botrytis cinerea*, and the bacterium *Xanthomonas citri,* with the goal of investigating the potential of our isolates as biological control agents. We performed disk diffusion assays for all of our bacterial isolates against the above phytopathogens, using the standard *Bacillus subtilis* 168 strain as a negative control, and the *Bacillus velezensis* QST713 strain, commercialized as a biological control agent [52], as a positive control (Figure 5).

**Figure 6:**
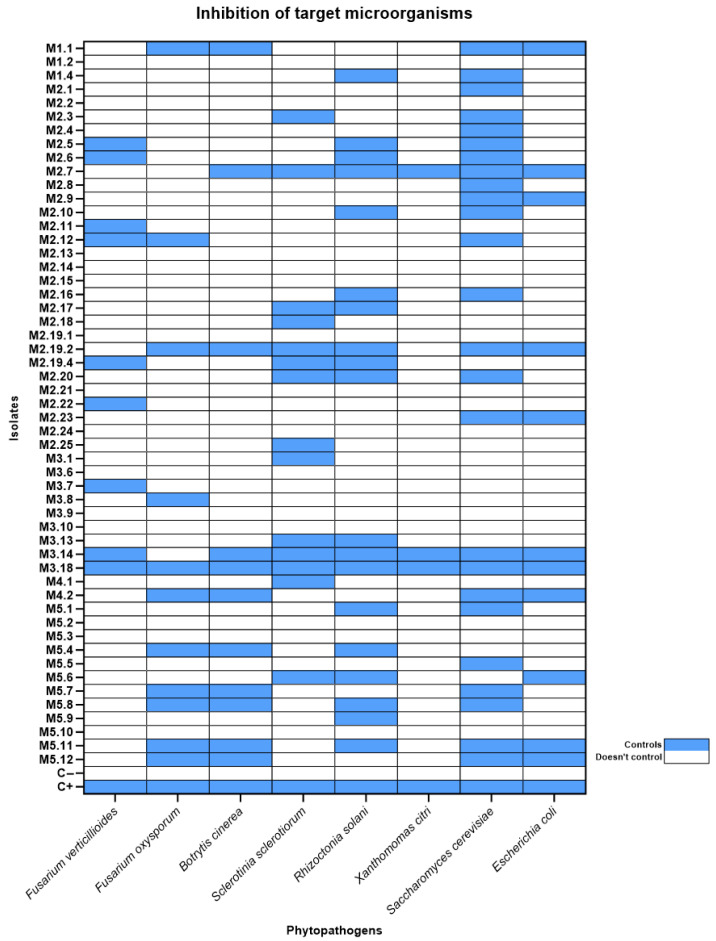
Inhibitory action of honey bacterial isolates against target organisms: *Fusarium verticillioides; F. oxysporum*; *Botrytis cinerea*; *Sclerotinia sclerotiorum*; *Rhizoctonia solani*; *Xanthomonas citri*; *Escherichia coli* DH5a; *Saccharomyces cerevisiae* BY4741/yEP-GAP-mCherry. The C+ indicates the commercial bacterial strain that was used as a positive control for the tests, and C− indicates the *B. subtillis* 168 strain used as a negative control.

Initial screening using *S. cerevisiae* BY4741/yEP-GAP-mCherry and *E. coli* DH5α proved to be effective in identifying isolates with promising antagonistic properties (Figure 4 and Figure 5). A strong correlation was observed between isolates that inhibited *S. cerevisiae* or *E. coli* and those active against the tested phytopathogens. This finding underscores the utility of these model organisms as cost-effective and rapid initial screening tools for identifying bioactive strains (Figure 6).

The results strongly support the use of proxy organisms for preliminary screening. Specifically, the capacity of isolates to inhibit the proxy organisms was a statistically significant predictor of broader antagonistic activity against phytopathogens. The Spearman’s rank correlation test indicated a moderate positive relationship between the proxy screening score, ranging from 0 proxy inhibited to 2 proxies inhibited, and the total number of inhibited phytopathogens (r_s_ = 0.4512, *p* = 0.0005). This confirms a robust monotonic association, where isolates inhibiting a higher number of proxies tend to control a greater number of phytopathogens. Furthermore, the Kruskal–Wallis test demonstrated a highly significant difference in the median number of inhibited phytopathogens across the three proxy score groups (H = 11.04, *p* = 0.004, Figure S1, Supplementary Materials). Subsequent Dunn’s post hoc test highlighted that the major difference lies between the extreme groups, isolates that inhibited both proxies controlled significantly a higher number of phytopathogens compared to those that did not inhibit both proxies (*p* = 0.0049). The linear trend confirms the moderate association found in Spearman’s correlation analysis.

Additionally, sensitivity and specificity metrics suggest that while the initial screening is useful, it requires optimization. The sensitivity (64.7%) and specificity (72.7%) values indicate that some true positives and true negatives are correctly identified, but a fraction of bioactive strains may be missed. To further evaluate predictive performance, a receiver operating characteristic (ROC) curve was generated (Figure S1, Supplementary Materials). The area under the curve (AUC = 0.6872) confirms the proxy’s capability for moderate discrimination between active and non-active isolates, though suggesting a potential for overlooking some bioactive strains (false negatives), highlighting the need for potential refinements.

These findings highlight the effectiveness of the current screening approach while emphasizing the need for refinement to maximize its applicability in microbial biocontrol discovery. Potential future enhancements may involve refining inhibition thresholds, incorporating additional model organisms, or integrating complementary assays to enhance predictive capability.

### 3.3. Fungal Inhibition and Morphological Analysis

**Figure 7:**
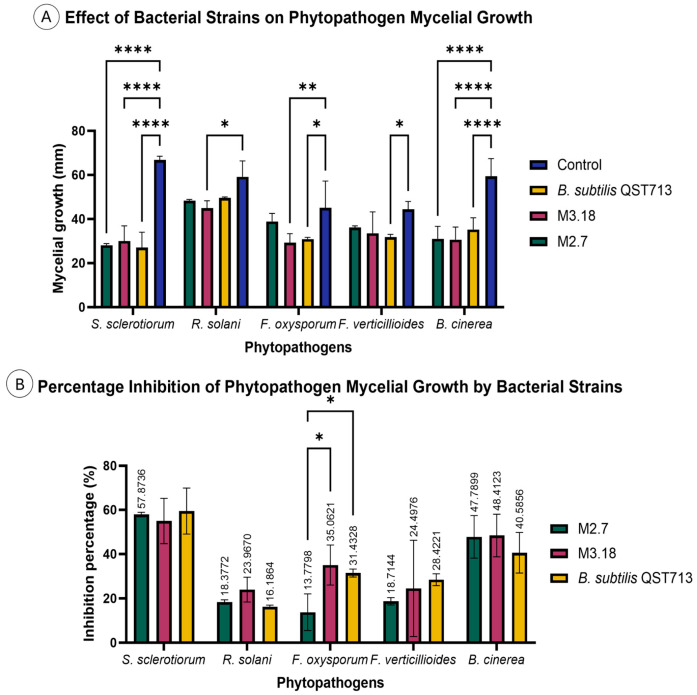
Antagonistic effect of selected bacterial strains on phytopathogen mycelial growth: (**A**) Raw measurements of mycelial hyphae. Bar graph illustrating the absolute mean length of phytopathogen hyphae (*Y*-axis, in mm) across the different treatments: untreated control, and confrontation with isolates M2.7, M3.18, and the commercial standard, *Bacillus subtilis* QST713. Error bars represent the standard error of the mean (SEM). Significant differences compared to the control are indicated by an asterisk (*). (**B**) Percentage inhibition of mycelial growth. Bar graph representing the antagonistic efficiency of the bacterial strains, calculated as the percentage reduction in mycelial growth relative to the untreated control. This panel effectively quantifies the inhibitory power of strains M2.7, M3.18, and *B. subtilis* QST713. Statistical significance compared to the untreated control is indicated by asterisks: (* *p* < 0.05), ** (*p* < 0.01), and **** (*p* < 0.0001). Error bars represent the standard error of the mean (SEM).

The two bacterial strains that had the strongest results in the qualitative tests, namely M3.18 and M2.7, were selected to be quantitatively tested. They demonstrated varying degrees of inhibitory activity against the selected phytopathogenic fungi in dual culture assays, in which inhibition zones were measured to quantify their antagonistic effect. The results for phytopathogen inhibition were evaluated by measuring fungal growth and calculating inhibition percentages (Figure 7), with all corresponding raw data provided in the Supplementary Material (Table S1). The results indicated significant differences in the growth rates of fungi in response to bacterial treatment.

Both bacterial strains M2.7 and M3.18 demonstrated a reduction in the growth radius of *R. solani* compared to the negative control, suggesting that these strains possess inhibitory effects on fungal growth. While the commercially available *B. velezensis* strain also reduced the growth radius of the fungus, the inhibitory effect was less pronounced than that of M3.18, indicating a potentially stronger biocontrol action by strain M3.18. Both M2.7 and M3.18 exhibited a strong inhibitory effect on *S. sclerotiorum* growth, with a significant reduction in growth radius compared to the negative control. However, the results between the novel bacterial strains and the commercially available strain were quite similar, indicating that M2.7 and M3.18 were comparably effective to the positive control.

The percentage of inhibition was calculated to quantify the extent to which the bacterial strains hindered fungal growth. The percentage of inhibition for both M2.7 and M3.18 was higher than that of the commercially available strain, indicating that the novel bacterial strains might offer superior biocontrol efficacy against *R. solani*. Of the two novel strains, M3.18 exhibited the highest inhibition, supporting its potential as a promising biocontrol agent for *R. solani*. Despite slight variations in the inhibition percentages, all three treatments exhibited strong antifungal effects, with inhibition percentages around 59%, underscoring the effectiveness of these strains in controlling *S. sclerotiorum* growth.

**Figure 8:**
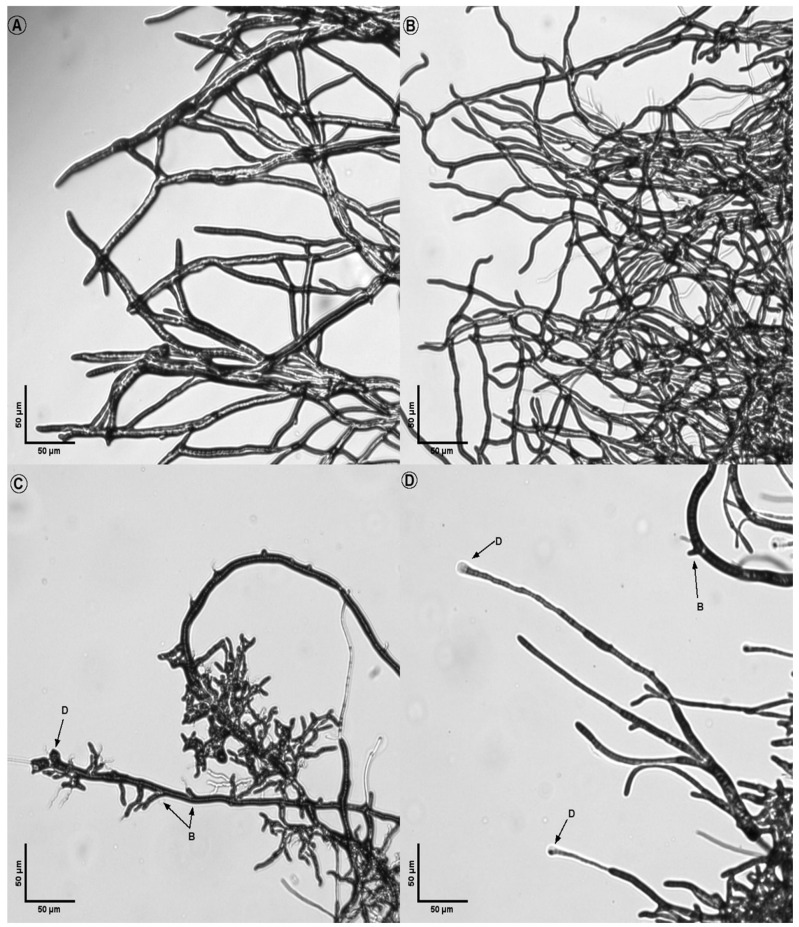
Inhibitory action of bacterial strains on *Fusarium verticillioides* hyphae: (**A**) *F. verticillioides* alone; (**B**) fungal hyphae in the presence of M2.7; (**C**) fungal hyphae in the presence of M3.18; (**D**) fungal hyphae in the presence of the *B. velezensis* commercial strain. Legend: D indicates defense structures; B indicates points of hyphal branching.

**Figure 9:**
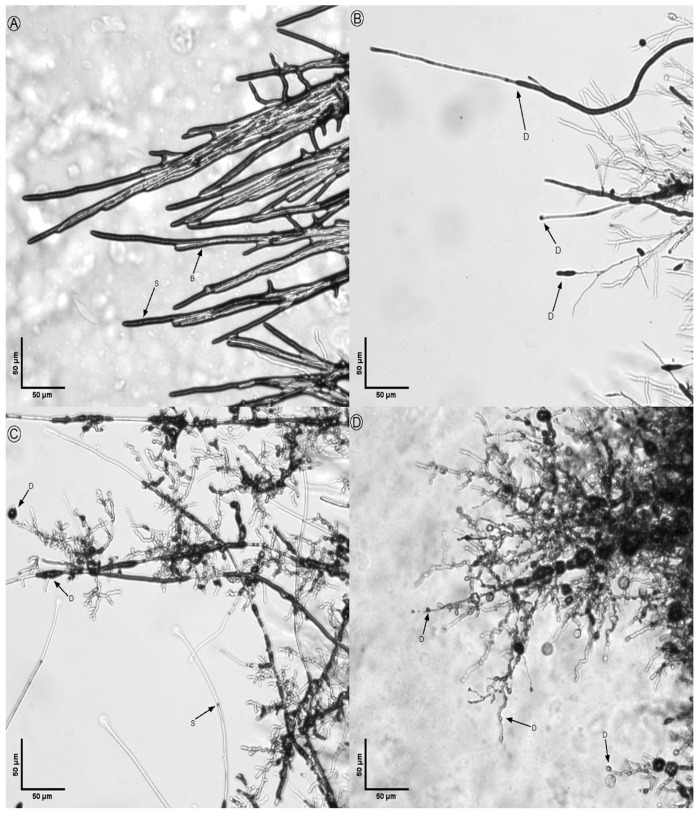
Inhibitory action of bacterial strains on *Fusarium oxysporum* hyphae: (**A**) *F. oxysporum* alone; (**B**) fungal hyphae in the presence of M2.7; (**C**) fungal hyphae in the presence of M3.18; (**D**) fungal hyphae in the presence of the *B. velezensis* commercial strain. Legend: D indicates defense structures; S indicates septa; B indicates points of hyphal branching.

**Figure 10:**
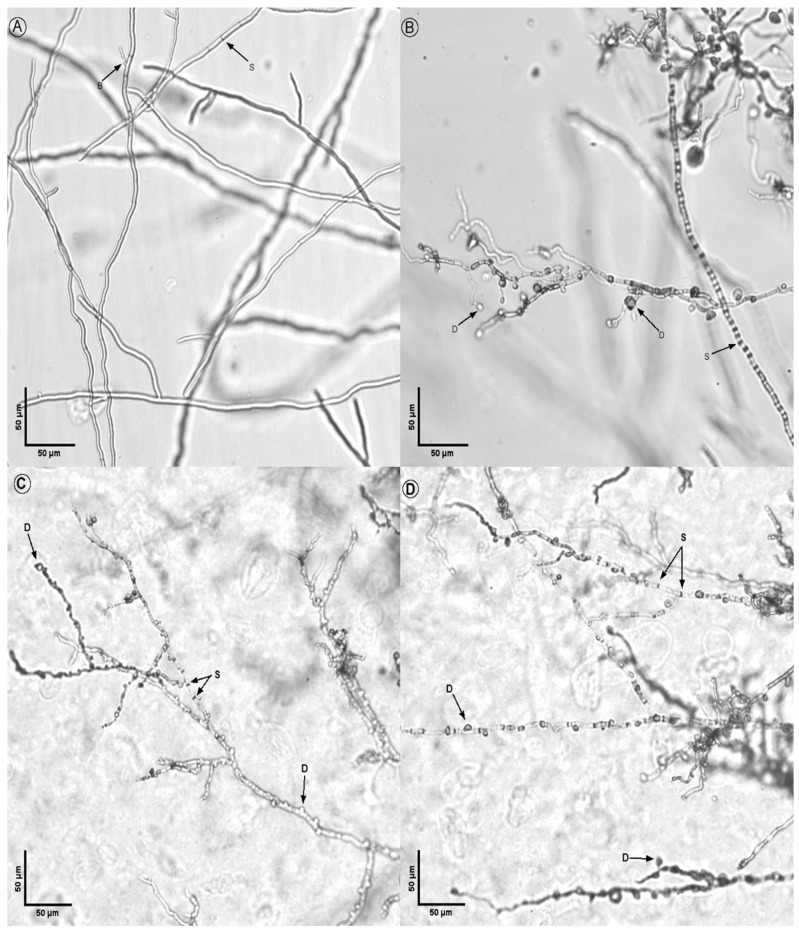
Inhibitory action of bacterial strains on *Botrytis cinerea* hyphae. (**A**) *B. cinerea* alone; (**B**) fungal hyphae in the presence of M2.7; (**C**) fungal hyphae in the presence of M3.18; (**D**) fungal hyphae in the presence of the *B. velezensis* commercial strain. Legend: D indicates defense structures; S indicates septa; B indicates points of hyphal branching.

**Figure 11:**
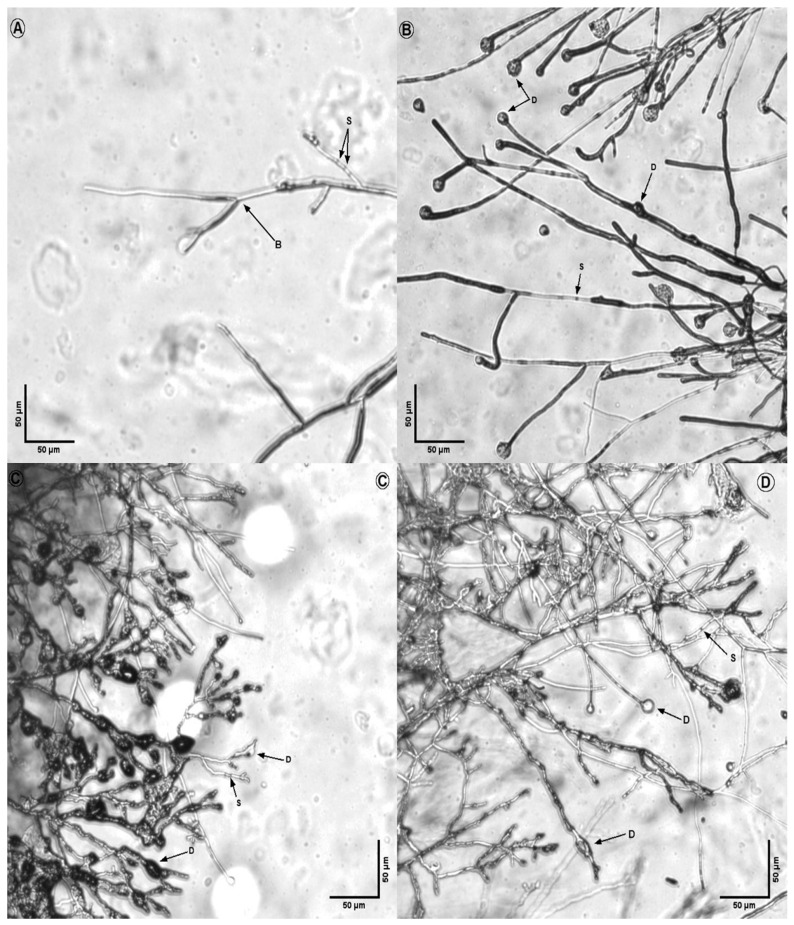
Inhibitory action of bacterial strains on *Sclerotinia sclerotiorum* hyphae: (**A**) *S. sclerotiorum* alone; (**B**) fungal hyphae in the presence of M2.7; (**C**) fungal hyphae in the presence of M3.18; (**D**) fungal hyphae in the presence of the *B. velezensis* commercial strain. Legend: D indicates defense structures; S indicates septa; B indicates points of hyphal branching.

**Figure 12:**
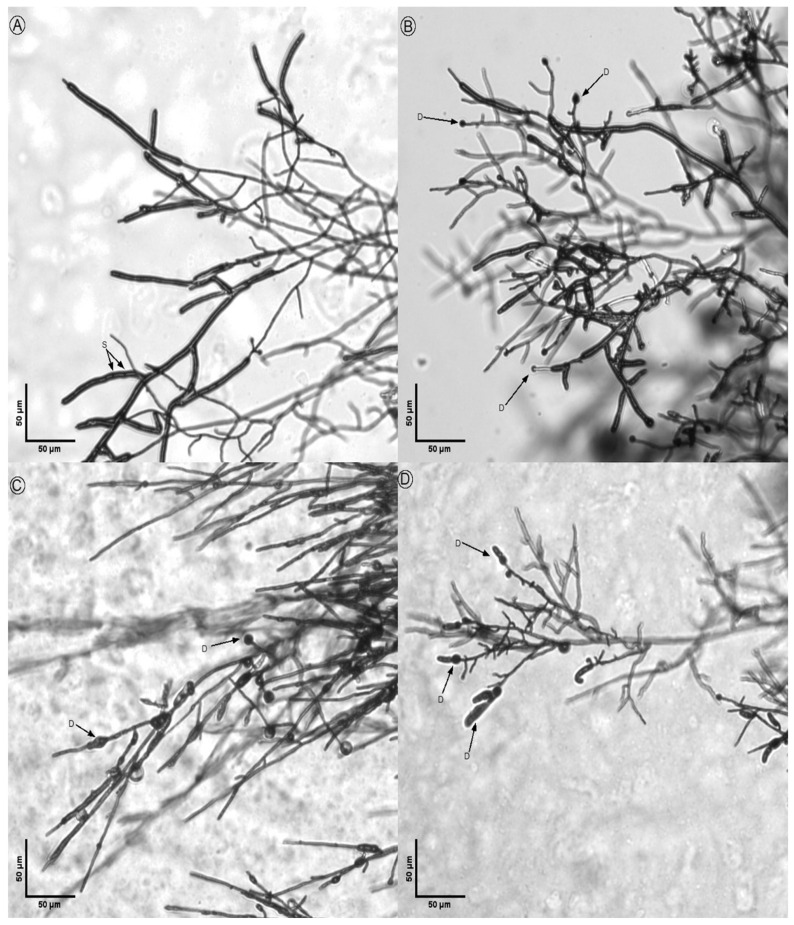
Inhibitory action of bacterial strains on *Rhizoctonia solani* hyphae: (**A**) *R. solani* alone; (**B**) fungal hyphae in the presence of M2.7; (**C**) fungal hyphae in the presence of M3.18; (**D**) fungal hyphae in the presence of the *B. velezensis* commercial strain. Legend: D indicates defense structures; S indicates septa.

The morphological analysis of treated fungi revealed significant alterations in hyphal structures compared to untreated controls (Figure 8, Figure 9, Figure 10, Figure 11 and Figure 12). Under light microscopy, fungal hyphae in untreated cultures appeared typical for each fungal species. *Fusarium verticillioides* displayed thin, elongated hyphae with regular septation and moderate branching, typical of this species (Figure 8), whereas *Fusarium oxysporum* showed dense, moderately branched hyphae with slightly wider diameters and distinct septation, reflecting its characteristic morphology (Figure 9). For *Botrytis cinerea*, the hyphae were long, cylindrical, and relatively unbranched, with sparse septation characteristic of optimal growth conditions (Figure 10), *Sclerotinia sclerotiorum* hyphae were hyaline, branched, and multinucleated (Figure 11), and *Rhizoctonia solani* exhibited wide, ribbon-like hyphae with multinucleate cells, minimal septation, and branching at right angles, all consistent with its normal growth (Figure 12).

Upon exposure to the bacterial strains M2.7, M3.18, and the commercially available *B. velezensis QST713*, significant morphological changes were observed across all fungal species. In *B. cinerea*, the hyphae exhibited noticeable deformities, including swelling at the tips, increased septation, and the formation of rounded defensive structures, possibly resembling appressoria. In *F. verticillioides*, treated hyphae displayed irregular swelling, increased branching, and distortions in their normally elongated shape. *F. oxysporum* hyphae showed twisting, fragmentation, and thickened regions, suggesting a heightened stress response. Similarly, *R. solani* exhibited pronounced swelling and fragmentation, with abnormal branching angles and the formation of stress-related structures.

Across all fungi, the hyphal deformities included twisting, swelling, and structural disruptions, indicating stress induced by the bacterial treatments. Increased septation (S), heightened branching (B), and the formation of defense structures (D), such as thickened cell walls or melanized regions, were observed. These morphological alterations suggest that the bacterial strains induce strong defensive responses in the fungi, likely through the secretion of antimicrobial compounds or other inhibitory mechanisms. The observed changes highlight the potential of the bacterial strains M2.7 and M3.18, as well as the commercial strain, to act as effective biocontrol agents by disrupting fungal growth and inducing structural defenses.

These alterations are consistent with previously observed responses of the phytopathogens to certain antagonistic bacteria, where the fungus undergoes metabolic shifts in response to the microbial interaction, possibly as a defensive mechanism.

### 3.4. Sequencing and Molecular Identification of Bacterial Strains

The bacterial strains were subjected to molecular and taxonomic identification using 16S rDNA sequencing and subsequent comparison with two databases: EZBioCloud 16S and NCBI BLASTn (Table 3). The results revealed a high degree of variability in identification accuracy at the species level between the two platforms. Despite this, all isolates were consistently identified at the genus level as belonging to the genus *Bacillus.*

Among the strains analyzed, species-level identification was often ambiguous. For example, strain M2.1 was identified as *B. altitudinis* with a 99.02% match in EZBioCloud, while BLASTn yielded a lower confidence match of 97.77%. Similar discrepancies were observed for other strains. Strain M2.5, for instance, was associated with multiple potential species in both databases, each achieving above 99% similarity, reflecting the inherent challenges in confidently resolving species-level taxonomy solely through 16S rDNA sequencing.

Interestingly, certain strains achieved a slightly higher degree of resolution. For instance, M3.18 was consistently identified as *B. velezensis* with 99.85% similarity in EZBioCloud and 99.7% in BLASTn. However, alternative closely related species such as *B. siamensis* and *B. amyloliquefaciens* were also proposed, illustrating the difficulty in differentiating closely related members of the *Bacillus subtilis* group using 16S rDNA alone.

The strains exhibiting significant antagonistic activity against phytopathogens, M2.7 and M3.18, were also analyzed using the gene *gyrA* to achieve a better resolution of species identification within these *Bacillus* strains. The sequencing was performed using two primer pairs, “gyrA1” and “gyrA2”, and the results are presented in Table 4.

For strain M2.7, sequencing with the primer pair “gyrA1” revealed a 99.7% similarity to both *Bacillus amyloliquefaciens* and *Bacillus velezensis*, while sequencing with “gyrA2” resulted in slightly higher similarity values of 99.75% for both species. Similarly, strain M3.18 exhibited 99.83% similarity to *Bacillus amyloliquefaciens* and *Bacillus velezensis* with the “gyrA1” primer pair, whereas the “gyrA2” primer pair yielded slightly lower similarity values of 99.09% for both species.

These findings reinforce the difficulty in differentiating closely related members of the *Bacillus subtilis* group solely based on 16S rDNA sequencing. The *gyrA* gene analysis provided a higher resolution but still did not allow for definitive species-level distinction between *Bacillus amyloliquefaciens* and *Bacillus velezensis*, as both strains exhibited near-identical sequence similarities to both species across both primer sets.

## 4. Discussion

### 4.1. Honey as a Reservoir for Stress-Tolerant Microorganisms and Efficiency of Proxies

This research elucidates honey as an environment that has received little scientific attention concerning the bioprospecting of microorganisms with potential for biocontrol, even though its unique chemical, biological, and ecological features—such as high osmolarity, intraspecific competition, and antibiosis—favor a rich microbiota that is resistant to stress and capable of producing secondary metabolites.

The initial bioprospecting phase aimed to screen commercial honey samples for culturable microorganisms, directly challenging the prevailing view of commercial honey as a microbially inhospitable environment due to its high sugar content, antimicrobial properties, and low water activity [53,54,55,56]. Also, the samples analyzed were commercial honeys, meaning they had undergone standard industrial processing, such as pasteurization and filtration. These treatments are typically employed to improve clarity, prevent crystallization, and they also reduce the microbial load to extend shelf life [57,58,59,60]. The finding of a rather large variety of microorganisms across most samples directly contradicts the effectiveness of commercial processing against some highly resilient, spore-forming, and osmotolerant microbes. This persistent diversity highlights the extreme survival capacity of the honey-associated microbial communities, which is a key trait for potential biocontrol agents.

The bioprospecting results highlighted significant variability in microbial colonization based on the honey source. Specifically, honeys from *Apis mellifera* with comb fragments (Honey 3) and from *Tetragonisca* sp., a Brazilian stingless bee (Honey 2), yielded the highest colony count. The elevated count in Honey 3 can be attributed to the comb fragments, which serve as a known protected microbial reservoir within the hive structure [61]. Similarly, the higher load in stingless bee honey could be linked to the unique microbial inoculation and fermentation processes that occur in the storage pots within the hive, or even the differences in post-harvesting processing. In contrast, the low counts in the commercial sachet honey and honeydew may reflect a greater impact of commercial processing. While the origin of the isolated microbiota cannot be definitively determined from these results alone, the findings confirm the value of even commercially treated honey as a promising environment for bioprospecting for novel, stress-tolerant microbial candidates.

The use of *S. cerevisiae* and *E. coli* DH5α as initial test organisms is a practical approach for large-scale evaluation of microbial strains. Both organisms were highly sensitive to bioactive compounds; most isolates that inhibited phytopathogens also affected one or both models, with only 20% of microbial isolates missed. This suggests that these model organisms can serve as efficient proxies for detecting strains with antimicrobial potential before testing against more labor-intensive and time-expensive phytopathogens. The simplicity, low cost, and reproducibility of using *S. cerevisiae* and *E. coli* further streamline the screening process, making it feasible to quickly narrow down thousands of isolates to the most promising candidates for biocontrol applications.

### 4.2. Comparative Efficacy and Morphological Interpretations

The quantitative analysis of isolates M2.7 and M3.18 reveals a competitive edge, particularly when observing the inhibitory effect on the growth of *R. solani* and *S. sclerotiorum* compared to the commercial benchmark *B. velezensis* QST713 (C+). M3.18 (~24%) had higher antifungal activity against *R. solani* than M2.7 (~18%) and the positive control C+ (~15%). In the case of *S. sclerotiorum*, the inhibition rates (reaching ~59% for M3.18) were statistically equivalent to the commercial product. Beyond these pathogens, both novel strains also demonstrated varying degrees of inhibitory activity against *B. cinerea*, *F. verticillioides*, and *F. oxysporum.*

Quantitative assays indicated that M2.7 and M3.18 consistently possess broad-spectrum antagonistic effects. These findings were corroborated by the morphological analysis of the treated hyphae, which revealed structural changes across all tested fungal species, including distortions in shape and irregular septation. The deformities indicate a possible stress induced response in the fungi, likely due to the secretion of antimicrobial compounds, highlighting the generalized disruptive mechanism of the bacterial strains against diverse fungal growth patterns.

The structural changes observed across all tested fungal species (Figure 8, Figure 9, Figure 10, Figure 11 and Figure 12) provide some insights into the possible modes of action of M2.7 and M3.18. The deformities found indicate significant physiological stress, specifically “bulbing” and fragmentation, which are classic indicators of cell wall disruption and osmotic imbalance [61,62,63]. In *Bacillus* species, such alterations are frequently associated with the secretion of lipopeptides (like iturins and fengycins), which target fungal membranes, or lytic enzymes that degrade cell walls [61,62,63]. The formation of defensive structures, such as melanized regions or thickened walls, further suggests that the pathogens are undergoing a heightened stress response.

### 4.3. Taxonomic Identification and Ecological Origin

Molecular characterization through *16S rDNA* and *gyrA* sequencing confirmed the identity of the strains, showing high genetic relatedness to microorganisms known for their capability to produce bioactive metabolites, such as *Bacillus velezensis* and *B. amyloliquefaciens*. The BLAST results pointed out that strains M2.7 and M3.18 are closely related to bacteria able to produce antimicrobial compounds, confirming their potential as promising biocontrol agents. *B. velezensis* is a well-known biocontrol species, and its isolation from commercial honey suggests a unique ecological resilience. These isolated strains show the capacity of surviving the extreme osmotic stress of honey, as well as industrial pasteurization, possessing a “pre-conditioning” that may make them resilient to environmental stressors in the field—such as desiccation and UV radiation—compared to the strains isolated from soil environments. Further studies are needed to identify the biosynthetic pathways responsible for their antimicrobial activities and also their efficacy and safety in a wide range of agricultural scenarios. Our findings suggest that bacteria isolated from food matrices can match or even exceed the performance of globally established commercial strains, underscoring their potential as high-performance candidates capable of competing in the current bio-input market. This study, therefore, contributes not only to biocontrol agent discovery but also underlines the potential of natural products—in this case, commercial honey—to be a valuable source of microbial diversity serving agricultural innovation.

## Figures and Tables

**Table 1:** Description of different types of honey and honeydew analyzed in the study.

Code	Type of Honey	Bee Species	Floral Source	Region
Honey 1	Honey sachet	*Apis mellifera*	Wildflowers	State of São Paulo
Honey 2	Honey	*Tetragonisca angustula*	Wildflowers	Not specified
Honey 3	Honey with comb	*Apis mellifera*	Wildflowers	Atibaia, São Paulo (SP)
Honey 4	Honeydew	*Apis mellifera*	*Mimosa scabrella* flowers	Southern Brazil
Honey 5	Honey	*Apis mellifera*	Wildflowers	Campinas, São Paulo (SP)

**Table 2:** List of primers for sequencing the 16S DNA gene and gyrase subunitA gene.

Code	Sequence
16S_Long F	TAAGACTGGGATAACTCCGG
16S_Long R	CCATTGTAGCACGTGTGTAG
gyrA1_F	CAGTCAGGAAATGCGTACGTCCTT
gyrA1_R	CAAGGTAATGCTCCAGGCATTGCT
gyrA2_F	GCDGCHGCNATGCGTTAYAC
gyrA2_R	ACAAGMTCWGCKATTTTTTC

**Table 3:** Taxonomic identification results of the aligned 16S DNA gene sequences of the bacterial isolates that demonstrated antimicrobial activity using EZBioCloud 16S database (https://www.ezbiocloud.net/; accessed on 14 November 2024) and NCBI BLASTn database (https://blast.ncbi.nlm.nih.gov/Blast.cgi; accessed on 14 November 2024).

Code	EzbioCloud		Blastn	
	Species	%	Species	%
M2.1	*Bacillus altitudinis*	99.02	*Bacillus altitudinis*	97.77
M2.2	*Bacillus licheniformis*	99.21	*Bacillus licheniformis*	99.12
M2.3	*Bacillus altitudinis*	99.46	*Bacillus stratosphericus*	99.06
	*Bacillus xiamenensis*	99.32	*Bacillus altitudinis*	99.06
M2.5	*Bacillus zhangzhouensis*	99.71	*Bacillus zhangzhouensis*	99.43
	*Bacillus safensis*	99.71	*Bacillus safensis*	99.43
	*Bacillus pumilus*	99.57	*Bacillus pumilus*	99.43
	*Bacillus australimaris*	99.57	*Bacillus australimaris*	99.43
	*Bacillus altitudinis*	99.14	*Bacillus altitudinis*	99.43
M2.6	*Bacillus altitudinis*	100	*Bacillus altitudinis*	99.56
M2.7	*Bacillus altitudinis*	100	*Bacillus pumilus*	99.73
	*Bacillus pumilus*	99.86	*Bacillus altitudinis*	99.59
M2.8	*Bacillus altitudinis*	98.94	*Bacillus altitudinis*	99.54
	*Bacillus xiamenensis*	98.79	*Bacillus aerophilus*	99.54
M2.10	*Bacillus zhangzhouensis*	99.54	*Bacillus zhangzhouensis*	99.1
	*Bacillus safensis*	99.54	*Bacillus safensis*	99.1
	*Bacillus pumilus*	99.39	*Bacillus pumilus*	99.1
M2.16	*Bacillus pumilus*	99.58	*Bacillus pumilus*	99.02
	*Bacillus zhangzhouensis*	99.58	*Bacillus zhangzhouensis*	99.02
	*Bacillus safensis*	99.58	*Bacillus safensis*	99.02
	*Bacillus australimaris*	99.43	*Bacillus australimaris*	99.02
M2.20	*Bacillus zhangzhouensis*	98.82	*Bacillus zhangzhouensis*	98.55
	*Bacillus safensis*	98.82	*Bacillus safensis*	98.55
	*Bacillus pumilus*	98.67	*Bacillus pumilus*	98.55
	*Bacillus australimaris*	98.67	*Bacillus australimaris*	98.4
M2.23	*Bacillus altitudinis*	97.83	*Bacillus altitudinis*	99.84
M2.24	*Bacillus thuringiensis*	99.07	*Bacillus thuringiensis*	99.21
	*Bacillus toyonensis*	99.07	*Bacillus toyonensis*	99.08
	*Bacillus proteolyticus*	99.07	*Bacillus proteolyticus*	99.08
M3.6	*Bacillus toyonensis*	99.69	*Bacillus toyonensis*	99.54
	*Bacillus wiedmannii*	99.69	*Bacillus wiedmannii*	99.54
	*Bacillus cereus*	99.54	*Bacillus cereus*	99.69
M3.18	*Bacillus velezensis*	99.85	*Bacillus velezensis*	99.7
	*Bacillus siamensis*	99.85	*Bacillus siamensis*	99.55
	*Bacillus amyloliquefaciens*	99.7	*Bacillus amyloliquefaciens*	99.7
M5.1	*Bacillus zhangzhouensis*	100	*Bacillus zhangzhouensis*	99.7
	*Bacillus safensis*	100	*Bacillus safensis*	99.7
	*Bacillus pumilus*	99.85	*Bacillus pumilus*	99.7
	*Bacillus australimaris*	99.85	*Bacillus australimaris*	99.55
M5.3	*Bacillus toyonensis*	98.99	*Bacillus toyonensis*	99.42
	*Bacillus wiedmannii*	98.99	*Bacillus wiedmannii*	99.28
	*Bacillus albus*	98.99	*Bacillus albus*	99.28
	*Bacillus proteolyticus*	98.99	*Bacillus proteolyticus*	99.28
	*Bacillus cereus*	98.84	*Bacillus cereus*	99.42
	*Bacillus thuringiensis*	98.99	*Bacillus thuringiensis*	99.28
M5.8	*Lysinibacillus fusiformis*	97.56	*Lysinibacillus fusiformis*	96
M5.11	*Lysinibacillus fusiformis*	99.45	*Lysinibacillus fusiformis*	99.45
	*Lysinibacillus sphaericus*	99.32	*Lysinibacillus sphaericus*	99.46

**Table 4:** Taxonomic identification results of the gyrase subunitA gene sequences of the bacterial isolates M2.7 and M3.18 utilizing NCBI BLASTn.

Code	Primer	Specie	
M2.7	gyrA1	*Bacillus amyloliquefaciens*	99.7
		*Bacillus velezensis*	99.7
	gyrA2	*Bacillus amyloliquefaciens*	99.75
		*Bacillus velezensis*	99.75
M3.18	gyrA1	*Bacillus amyloliquefaciens*	99.83
		*Bacillus velezensis*	99.83
	gyrA2	*Bacillus amyloliquefaciens*	99.09
		*Bacillus velezensis*	99.09

## Data Availability

The original contributions presented in this study are included in the article. Further inquiries can be directed to the corresponding author.

## Supplementary Materials

The following supporting information can be downloaded at: https://www.mdpi.com/article/10.3390/microorganisms14010224/s1, Figure S1: Validation of the proxy inhibition score as a predictor of antagonistic activity against phytopathogens; Table S1: Values of fungal inhibition of the dual culture assays.

## References

[1] Maitra S., Brestic M., Bhadra P., Shankar T., Praharaj S., Palai J.B., Shah M.M.R., Barek V., Ondrisik P., Skalický M. (2022). Bioinoculants—Natural Biological Resources for Sustainable Plant Production. Microorganisms.

[2] Chakraborty S., Newton A.C. (2011). Climate Change, Plant Diseases and Food Security: An Overview. Plant Pathol..

[3] Miller S.A., Ferreira J.P., LeJeune J.T. (2022). Antimicrobial Use and Resistance in Plant Agriculture: A One Health Perspective. Agriculture.

[4] Kelbrick M., Hesse E., O’ Brien S. (2023). Cultivating Antimicrobial Resistance: How Intensive Agriculture Ploughs the Way for Antibiotic Resistance. Microbiology.

[5] FAO (2022). Pesticides Use, Pesticides Trade and Pesticides Indicators.

[6] John D.A., Babu G.R. (2021). Lessons from the Aftermaths of Green Revolution on Food System and Health. Front. Sustain. Food Syst..

[7] Brunelle T., Chakir R., Carpentier A., Dorin B., Goll D., Guilpart N., Maggi F., Makowski D., Nesme T., Roosen J. (2024). Reducing Chemical Inputs in Agriculture Requires a System Change. Commun. Earth Environ..

[8] Batuman O., Britt-Ugartemendia K., Kunwar S., Yilmaz S., Fessler L., Redondo A., Chumachenko K., Chakravarty S., Wade T. (2024). The Use and Impact of Antibiotics in Plant Agriculture: A Review. Phytopathology.

[9] Pathak V.M., Verma V.K., Rawat B.S., Kaur B., Babu N., Sharma A., Dewali S., Yadav M., Kumari R., Singh S. (2022). Current Status of Pesticide Effects on Environment, Human Health and It’s Eco-Friendly Management as Bioremediation: A Comprehensive Review. Front. Microbiol..

[10] McKernan C., Benson T., Farrell S., Dean M. (2021). Antimicrobial Use in Agriculture: Critical Review of the Factors Influencing Behaviour. JAC-Antimicrob. Resist..

[11] Verhaegen M., Bergot T., Liebana E., Stancanelli G., Streissl F., Mingeot-Leclercq M.-P., Mahillon J., Bragard C. (2023). On the Use of Antibiotics to Control Plant Pathogenic Bacteria: A Genetic and Genomic Perspective. Front. Microbiol..

[12] Fisher M.C., Alastruey-Izquierdo A., Berman J., Bicanic T., Bignell E.M., Bowyer P., Bromley M., Brüggemann R., Garber G., Cornely O.A. (2022). Tackling the Emerging Threat of Antifungal Resistance to Human Health. Nat. Rev. Microbiol..

[13] Mansfield J., Genin S., Magori S., Citovsky V., Sriariyanum M., Ronald P., Dow M., Verdier V., Beer S.V., Machado M.A. (2012). Top 10 Plant Pathogenic Bacteria in Molecular Plant Pathology. Mol. Plant. Pathol..

[14] Starr M.P., Chatterjee A.K. (1972). The Genus Erwinia: Enterobacteria Pathogenic to Plants and Animals. Annu. Rev. Microbiol..

[15] Timilsina S., Potnis N., Newberry E.A., Liyanapathiranage P., Iruegas-Bocardo F., White F.F., Goss E.M., Jones J.B. (2020). Xanthomonas Diversity, Virulence and Plant–Pathogen Interactions. Nat. Rev. Microbiol..

[16] Brunings A.M., Gabriel D.W. (2003). *Xanthomonas citri*: Breaking the Surface. Mol. Plant. Pathol..

[17] Volkers R.J.M., van Doorn B.B.J.A., Blom N.I., van de Bilt J.L.J., Gorkink-Smits P.M.A., Landman M.N.M., Teunissen M., Pel M.J.C., Raaymakers T.M., Bergsma-Vlami M. (2024). *Xanthomonas citri* Pv. *citri* Findings in Citrus Fruits Imported in the Netherlands. Plant Health Prog..

[18] Haag T., Nicholson A., Ashby E., National Academies of Sciences, Engineering, and Medicine, Health and Medicine Division, Board on Global Health, Forum on Microbial Threats (2023). Fungicide Resistance in Plant Protection Use. The Role of Plant Agricultural Practices on Development of Antimicrobial Resistant Fungi Affecting Human Health: Proceedings of a Workshop Series.

[19] van Rhijn N., Storer I.S.R., Birch M., Oliver J.D., Bottery M.J., Bromley M.J. (2024). Aspergillus Fumigatus Strains That Evolve Resistance to the Agrochemical Fungicide Ipflufenoquin in Vitro Are Also Resistant to Olorofim. Nat. Microbiol..

[20] Hossain M.M., Sultana F., Li W., Tran L.-S.P., Mostofa M.G. (2023). *Sclerotinia sclerotiorum* (Lib.) de Bary: Insights into the Pathogenomic Features of a Global Pathogen. Cells.

[21] Akber M.A., Mubeen M., Sohail M.A., Khan S.W., Solanki M.K., Khalid R., Abbas A., Divvela P.K., Zhou L. (2023). Global Distribution, Traditional and Modern Detection, Diagnostic, and Management Approaches of Rhizoctonia Solani Associated with Legume Crops. Front. Microbiol..

[22] Ajayi-Oyetunde O.O., Bradley C.A. (2018). *Rhizoctonia solani*: Taxonomy, Population Biology and Management of Rhizoctonia Seedling Disease of Soybean. Plant Pathol..

[23] Blacutt A.A., Gold S.E., Voss K.A., Gao M., Glenn A.E. (2018). *Fusarium verticillioides*: Advancements in Understanding the Toxicity, Virulence, and Niche Adaptations of a Model Mycotoxigenic Pathogen of Maize. Phytopathology.

[24] Dong S., Jiang K., Huai B., Ye L., You J., Ma Y., Tan G. (2023). Genetic Variability and Pathogenicity of *Fusarium verticillioides* Isolates from the Summer-Sown Maize Regions in China. Plant Pathol..

[25] Shah D.A., De Wolf E.D., Paul P.A., Madden L.V. (2019). Functional Data Analysis of Weather Variables Linked to Fusarium Head Blight Epidemics in the United States. Phytopathology.

[26] Jackson E., Li J., Weerasinghe T., Li X. (2024). The Ubiquitous Wilt-Inducing Pathogen *Fusarium oxysporum*—A Review of Genes Studied with Mutant Analysis. Pathogens.

[27] Dongzhen F., Xilin L., Xiaorong C., Wenwu Y., Yunlu H., Yi C., Jia C., Zhimin L., Litao G., Tuhong W. (2020). *Fusarium* Species and *Fusarium oxysporum* Species Complex Genotypes Associated with Yam Wilt in South-Central China. Front. Microbiol..

[28] Kema G.H.J., Drenth A., Dita M., Jansen K., Vellema S., Stoorvogel J.J. (2021). Editorial: Fusarium Wilt of Banana, a Recurring Threat to Global Banana Production. Front. Plant Sci..

[29] Bi K., Liang Y., Mengiste T., Sharon A. (2023). Killing Softly: A Roadmap of Botrytis Cinerea Pathogenicity. Trends Plant Sci..

[30] Chen L. (2017). Complete Genome Sequence of *Bacillus velezensis* LM2303, a Biocontrol Strain Isolated from the Dung of Wild Yak Inhabited Qinghai-Tibet Plateau. J. Biotechnol..

[31] Cheung N., Tian L., Liu X., Li X. (2020). The Destructive Fungal Pathogen Botrytis Cinerea—Insights from Genes Studied with Mutant Analysis. Pathogens.

[32] Mann A., Nehra K., Rana J.S., Dahiya T. (2021). Antibiotic Resistance in Agriculture: Perspectives on Upcoming Strategies to Overcome Upsurge in Resistance. Curr. Res. Microb. Sci..

[33] Saini M., Saharan B.S., Kumar S., Badoni P., Jabborova D., Duhan J.S., Kamal N. (2024). Exploring Plant and Microbial Antimicrobials for Sustainable Public Health and Environmental Preservation. Discov. Public Health.

[34] Singh B.K., Delgado-Baquerizo M., Egidi E., Guirado E., Leach J.E., Liu H., Trivedi P. (2023). Climate Change Impacts on Plant Pathogens, Food Security and Paths Forward. Nat. Rev. Microbiol..

[35] Islam T., Rabbee M.F., Choi J., Baek K.-H. (2022). Biosynthesis, Molecular Regulation, and Application of Bacilysin Produced by Bacillus Species. Metabolites.

[36] Rabbee M.F., Ali M.S., Choi J., Hwang B.S., Jeong S.C., Baek K. (2019). *Bacillus velezensis*: A Valuable Member of Bioactive Molecules within Plant Microbiomes. Molecules.

[37] Cars O., Chandy S.J., Mpundu M., Peralta A.Q., Zorzet A., So A.D. (2021). Resetting the Agenda for Antibiotic Resistance through a Health Systems Perspective. Lancet Glob. Health.

[38] de Oliveira C.A., de Paula G.T., Pupo M.T. (2023). Produtos Naturais Bioativos a Partir da Associação Simbiótica de Abelhas e Bactérias: Bioactive Natural Products from the Symbiotic Association of Bees and Bacteria. Rev. Virtual Química.

[39] Grundmann C.O., Guzman J., Vilcinskas A., Pupo M.T. (2024). The Insect Microbiome Is a Vast Source of Bioactive Small Molecules. Nat. Prod. Rep..

[40] Dharampal P.S., Diaz-Garcia L., Haase M.A.B., Zalapa J., Currie C.R., Hittinger C.T., Steffan S.A. (2020). Microbial Diversity Associated with the Pollen Stores of Captive-Bred Bumble Bee Colonies. Insects.

[41] Shi C., Chen T., Lan C., Gan R., Yu J., Zhao F., Liu Y. (2024). iMetaOmics: Advancing Human and Environmental Health through Integrated Meta-omics. iMetaOmics.

[42] Schaffner U., Heimpel G.E., Mills N.J., Muriithi B.W., Thomas M.B., Gc Y.D., Wyckhuys K.A.G. (2024). Biological Control for One Health. Sci. Total Environ..

[43] Etesami H., Jeong B.R., Glick B.R. (2023). Biocontrol of Plant Diseases by *Bacillus* spp. *Physiol*. Mol. Plant Pathol..

[44] Akinsemolu A.A., Onyeaka H., Odion S., Adebanjo I. (2024). Exploring Bacillus Subtilis: Ecology, Biotechnological Applications, and Future Prospects. J. Basic Microbiol..

[45] Roussin-Léveillée C., Rossi C.A.M., Castroverde C.D.M., Moffett P. (2024). The Plant Disease Triangle Facing Climate Change: A Molecular Perspective. Trends Plant Sci..

[46] Sinacori M., Francesca N., Alfonzo A., Cruciata M., Sannino C., Settanni L., Moschetti G. (2014). Cultivable Microorganisms Associated with Honeys of Different Geographical and Botanical Origin. Food Microbiol..

[47] Bilsland E., Sparkes A., Williams K., Moss H.J., de Clare M., Pir P., Rowland J., Aubrey W., Pateman R., Young M. (2013). Yeast-Based Automated High-Throughput Screens to Identify Anti-Parasitic Lead Compounds. Open Biol..

[48] Edgington L.V., Khew K.L., Barron G.L. (1971). Fungitoxic Spectrum of Benzimidazole Compounds. Phytopathology.

[49] Camacho C., Coulouris G., Avagyan V., Ma N., Papadopoulos J., Bealer K., Madden T.L. (2009). BLAST+: Architecture and Applications. BMC Bioinform..

[50] Stavropoulou E., Remmas N., Voidarou C., Vrioni G., Konstantinidis T., Ntougias S., Tsakris A. (2023). Microbial Community Structure among Honey Samples of Different Pollen Origin. Antibiotics.

[51] Donkersley P., Rhodes G., Pickup R.W., Jones K.C., Wilson K. (2018). Bacterial Communities Associated with Honeybee Food Stores Are Correlated with Land Use. Ecol. Evol..

[52] Anastassiadou M., Arena M., Auteri D., Brancato A., Bura L., Carrasco Cabrera L., Chaideftou E., Chiusolo A., Crivellente F., European Food Safety Authority (2021). Peer Review of the Pesticide Risk Assessment of the Active Substance *Bacillus amyloliquefaciens* Strain QST 713 (Formerly *Bacillus subtilis* Strain QST 713). EFSA J..

[53] Almasaudi S.B., Al-Nahari A.A.M., Abd El-Ghany E.S.M., Barbour E., Al Muhayawi S.M., Al-Jaouni S., Azhar E., Qari M., Qari Y.A., Harakeh S. (2017). Antimicrobial Effect of Different Types of Honey on *Staphylococcus aureus*. Saudi J. Biol. Sci..

[54] Martinotti S., Laforenza U., Patrone M., Moccia F., Ranzato E. (2019). Honey-Mediated Wound Healing: H_2_O_2_ Entry through AQP3 Determines Extracellular Ca^2+^ Influx. Int. J. Mol. Sci..

[55] Alvarez-Suarez J.M., Gasparrini M., Forbes-Hernández T.Y., Mazzoni L., Giampieri F. (2014). The Composition and Biological Activity of Honey: A Focus on Manuka Honey. Foods.

[56] Bonsignore G., Martinotti S., Ranzato E. (2024). Honey Bioactive Molecules: There Is a World Beyond the Sugars. BioTech.

[57] Singh I., Singh S. (2018). Honey Moisture Reduction and Its Quality. J. Food Sci. Technol..

[58] Scepankova H., Pinto C.A., Paula V., Estevinho L.M., Saraiva J.A. (2021). Conventional and Emergent Technologies for Honey Processing: A Perspective on Microbiological Safety, Bioactivity, and Quality. Compr. Rev. Food Sci. Food Saf..

[59] Subramanian R., Umesh Hebbar H., Rastogi N.K. (2007). Processing of Honey: A Review. Int. J. Food Prop..

[60] Beux M.R., Ávila S., Surek M., Bordin K., Leobet J., Barbieri F., Ferreira S.M.R., Rosa E.A.R. (2022). Microbial Biodiversity in Honey and Pollen Pots Produced by *Tetragonisca angustula* (Jataí). Braz. Arch. Biol. Technol..

[61] Mantri B.U., Vahidinasab M., Berensmeier S. (2025). Engineering Strategies for Fungal Cell Disruption in Biotechnological Applications. Eng. Life Sci..

[62] Chen M., Wang H., Zhang C., Zhang Y., Sun H., Yu L., Zhang Q., Chen X., Xiao H. (2025). Recent Advances in Antimicrobial Lipopeptide Fengycin Secreted by *Bacillus*: Structure, Biosynthesis, Antifungal Mechanisms, and Potential Application in Food Preservation. Food Chem..

[63] Gao Z., Zhang B., Liu H., Han J., Zhang Y. (2017). Identification of Endophytic *Bacillus velezensis* ZSY-1 Strain and Antifungal Activity of Its Volatile Compounds against *Alternaria solani* and *Botrytis cinerea*. Biol. Control.

