# Supplementary material for: Bioprospecting Honey-Derived Microorganisms for the Biological Control of Phytopathogens
Source: Microorganisms. 2026 Jan 18;14(1):224. doi: 10.3390/microorganisms14010224 (PMC12844432; doi:10.3390/microorganisms14010224)
Supplement: Supplementary file 1 [file microorganisms-14-00224-s001.zip › microorganisms-4070284-supplementary.pdf]

Supplementary Material

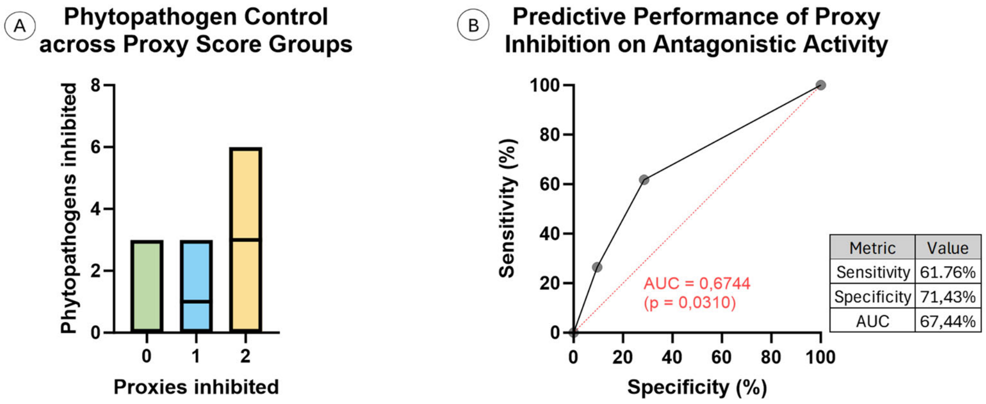

Figure S1. Validation of the proxy inhibition score as a predictor of antagonistic activity against phytopathogens. (a) Distribution of Total Antagonistic Activity by Proxy Inhibition Score. Box Plot showing the median number of inhibited phytopathogens for isolates grouped by the number of proxies controlled (0, 1, or 2). Median differences were statistically significant (Kruskal-Wallis $p = 0.0040$), confirming the positive monotonic relationship found by Spearman's correlation ($r_s = 0.4512$, $p = 0.0005$); (b) Predictive Performance of Proxy System (ROC Curve). Receiver Operating Characteristic (ROC) curve evaluating the proxy system's ability to discriminate between active and non-active isolates. The Area Under the Curve (AUC) of 0.6744 confirms the proxy's significant discriminatory power ($p = 0.0310$).

Table S1. Values of fungal inhibition of the dual culture assays.

Phytopathogen	Replicates	Measurements in mm			
		**M2.7**	**M3.18**	**C+**	**C-**
Rhizoctonia solani	I	47,613	45,691	49,466	56,478
	II	48,621	47,88	50,11	53,686
	III	48,662	41,402	49,209	67,355
	**Mean**	**48,299**	**44,991**	**49,595**	**59,173**
	**I %**	**18,377**	**23,967**	**16,186**	-
Sclerotinia sclerotiorum	I	28,553	22,437	35,065	68,074
	II	27,324	35,685	23,743	67,426
	III	28,485	32,036	22,382	64,759
	**Mean**	**28,121**	**30,053**	**27,063**	**66,753**
	**I %**	**57,873**	**54,979**	**59,457**	-
Fusarium oxysporum	I	42,525	31,679	31,733	36,356
	II	39,016	24,58	30,884	39,974
	III	35,083	31,578	30,129	58,933
	**Mean**	**38,875**	**29,279**	**30,915**	**45,088**
	**I %**	**13,780**	**35,062**	**31,433**	-
Fusarium verticillioides	I	36,594	27,222	33,101	43,863
	II	35,285	28,777	30,763	41,285
	III	36,53	44,697	31,598	48,22
	**Mean**	**36,136**	**33,565**	**31,821**	**44,456**
	**I %**	**18,714**	**24,498**	**28,422**	-

Phytopathogen	Replicates	Measurements in mm			
		M2.7	M3.18	C+	C-
Rhizoctonia solani	I	47,613	45,691	49,466	56,478
	II	48,621	47,88	50,11	53,686
	III	48,662	41,402	49,209	67,355
	**Mean**	**48,299**	**44,991**	**49,595**	**59,173**
	**I %**	**18,377**	**23,967**	**16,186**	-
Botrytis cinerea	I	36,409	27,577	39,428	62,088
	II	31,599	37,23	36,97	50,386
	III	25,02	27,112	29,206	65,706
	**Mean**	**31,009**	**30,640**	**35,201**	**59,393**
	**I %**	**47,790**	**48,412**	**40,732**	-

The test was made with isolates M2.7 and M3.18 and the commercial strain of *B. velezensis* QST713, as a positive control (C+), against five phytopathogenic fungi: *Rhizoctonia solani, Sclerotinia sclerotiorum, Fusarium oxysporum, Fusarium verticillioides* and *Botrytis cinerea*. The negative control plates (C-) were done plating the respective fungi without any bacterial isolate. All experiments were performed in triplicates.
